# Silibinin enhances the repair of ultraviolet B-induced DNA damage by activating p53-dependent nucleotide excision repair mechanism in human dermal fibroblasts

**DOI:** 10.18632/oncotarget.5519

**Published:** 2015-10-03

**Authors:** Ruth Guillermo-Lagae, Gagan Deep, Harold Ting, Chapla Agarwal, Rajesh Agarwal

**Affiliations:** ^1^ Department of Pharmaceutical Sciences, Skaggs School of Pharmacy and Pharmaceutical Sciences, Aurora, Colorado, USA; ^2^ University of Colorado Cancer Center, University of Colorado Denver, Aurora, Colorado, USA

**Keywords:** ultraviolet radiation B, photodamage, nucleotide excision repair, p53, silibinin

## Abstract

Ultraviolet radiation B (UVB) is the main cause of DNA damage in epidermal cells; and if not repaired, this DNA damage leads to skin cancer. In earlier studies, we have reported that natural flavonolignan silibinin exerts strong chemopreventive efficacy against UVB-induced skin damage and carcinogenesis; however mechanistic studies are still being actively pursued. Here, we investigated the role of nucleotide excision repair (NER) pathway in silibinin's efficacy to repair UVB-induced DNA damage. Normal human dermal fibroblasts (NHDFs) were exposed to UVB (1 mJ/cm^2^) with pre- or post- silibinin (100 μM) treatment, and cyclobutane pyrimidine dimers (CPDs) formation/repair was measured. Results showed that post-UVB silibinin treatment accelerates DNA repair via activating the NER pathway including the expression of XPA (xeroderma pigmentosum complementation group A), XPB, XPC, and XPG. In UVB exposed fibroblasts, silibinin treatment also increased p53 and GADD45α expression; the key regulators of the NER pathway and DNA repair. Consistently, post-UVB silibinin treatment increased the mRNA transcripts of XPA and GADD45α. Importantly, silibinin showed no effect on UVB-induced DNA damage repair in XPA- and XPB-deficient human dermal fibroblasts suggesting their key role in silibinin-mediated DNA damage repair. Moreover, in the presence of pifithrin-α, an inhibitor of p53, the DNA repair efficacy of silibinin was compromised associated with a reduction in XPA and GADD45α transcripts. Together, these findings suggest that silibinin's efficacy against UVB-induced photodamage is primarily by inhibiting NER and p53; and these findings further support silibinin's usage as a potential inexpensive, effective, and non-toxic agent for skin cancer chemoprevention.

## INTRODUCTION

Skin cancer is the most common cancer with more than 3 million cases diagnosed annually in the United States. This is roughly equivalent to the annual incidence of all other malignancies combined. Basal cell carcinoma (BCC) and squamous cell carcinoma (SCC), together known as non-melanoma skin cancers (NMSCs), account for about 95% of all skin cancer cases in the United States. Epidemiological and molecular data strongly suggest that more than 90% of NMSCs are associated with excessive exposure to solar ultraviolet B (UVB) (290–320 nm) radiation [1]. The primary lesions caused by UVB are cyclobutane pyrimidine dimers (CPDs) and 6–4 photoproducts (6–4 PPs). These lesions, if not repaired or removed, could give rise to mutations and cancer initiation. Subsequent selection and multiplication of these initiated cells results in further accumulation of mutations, ultimately leading to the development of skin tumors [2].

UVB-induced DNA damage is mainly repaired by the nucleotide excision repair (NER) pathway [3–5]. NER operates by global genomic repair (GGR), which removes lesions genome-wide, and transcription-coupled repair (TCR), which removes lesions specifically from DNA strands at actively transcribing genes. While the initial damage recognition is different in these two pathways, both pathways involve similar subsequent steps of excision of short damage-containing DNA segments, repair synthesis of a new DNA strand and finally DNA ligation of the new strand to the parental strand [4]. Defects in NER are associated with several human autosomal recessive hereditary disorders such as xeroderma pigmentosum (XP) and Cockayne's syndrome (CS) [3, 4]. The National Institutes of Health in a four decade follow up study found that in 65% of XP patients with skin cancer, NMSCs risk was increased 10,000-fold and melanoma risk was increased 2000-fold in patients under age 20 [6]. Overall, patients suffering from XP and CS exhibit extreme sensitivity to sun exposure and a marked predisposition to skin cancer, which clearly suggests an important role for DNA repair genes in the etiology of skin cancer.

NER is a complex process and requires more than 20 proteins with different functions in order to repair DNA [4, 7, 8]. First, CPDs and 6–4 PPs are recognized by protein complexes including the XPE and XPC gene products. After recognizing a lesion, TFIIH (transcription factor IIH) is recruited by XPC to open the DNA helix around the damage site. TFIIH is a multiprotein complex with several enzymatic activities including helicase (XPB and XPD) and kinase (cdk7) functions [4, 9]. Both helicases allow the unwinding of DNA upon which, the three dimensional structure is able to recruit XPA and RPA which are believed to join the TFIIH-repair complex to verify the nature of DNA structural alteration [4, 10]. Next, two endonucleases XPF-ERCC1 and XPG are recruited to the damage site to cleave damage-containing DNA, leading to the excision of ~24–32 nucleotides [4, 11]. Finally, DNA structure is restored by subsequent gap-filling DNA re-synthesis with the aid of auxiliary factors like PCNA [12]. Importantly, the NER repair machinery is inhibited by UVB-exposure [13], and unrepaired DNA photoproducts cause specific mutations (UVB-signature) in susceptible genes. For example, UVB-signature mutation in the p53 tumor suppressor gene is the most common event in SCC development. The mutational inactivation of p53 compromises NER, as p53 normally regulates the expression of several NER molecules and initiates key events during NER [14–18]. Consistently, loss of p53 in human cells results in reduced repair of UV-induced DNA damage, as observed in Li-Fraumeni syndrome patients [14, 17]. Therefore, non-toxic agent/s that activate both p53 and NER machinery could be important towards reducing UVB-induced photodamage and skin carcinogenesis.

Silibinin is a bioactive flavonolignan present in milk thistle (*Silybum marianum*) that has shown chemopreventive efficacy against several malignancies including skin cancer, as well as hepatoprotective, antioxidant and anti-inflammatory effects [19, 20]. Silibinin has a long history of use in traditional medicine systems for the treatment of several liver disorders [20]. Previous studies from our laboratory have clearly shown that silibinin reduces UVB-induced photodamage including CPDs formation by activating p53 in epidermal cells both in cell culture and animal models [21–23]. However, silibinin's effect on NER pathway especially in the context of silibinin-induced activation of p53 has not yet been studied. Results from the present study provide concrete evidence that silibinin promotes UVB-induced DNA damage repair via activating NER machinery and p53 is critical therein.

## RESULTS

### Silibinin repairs UVB-induced DNA damage in NHDFs

**Figure 1:**
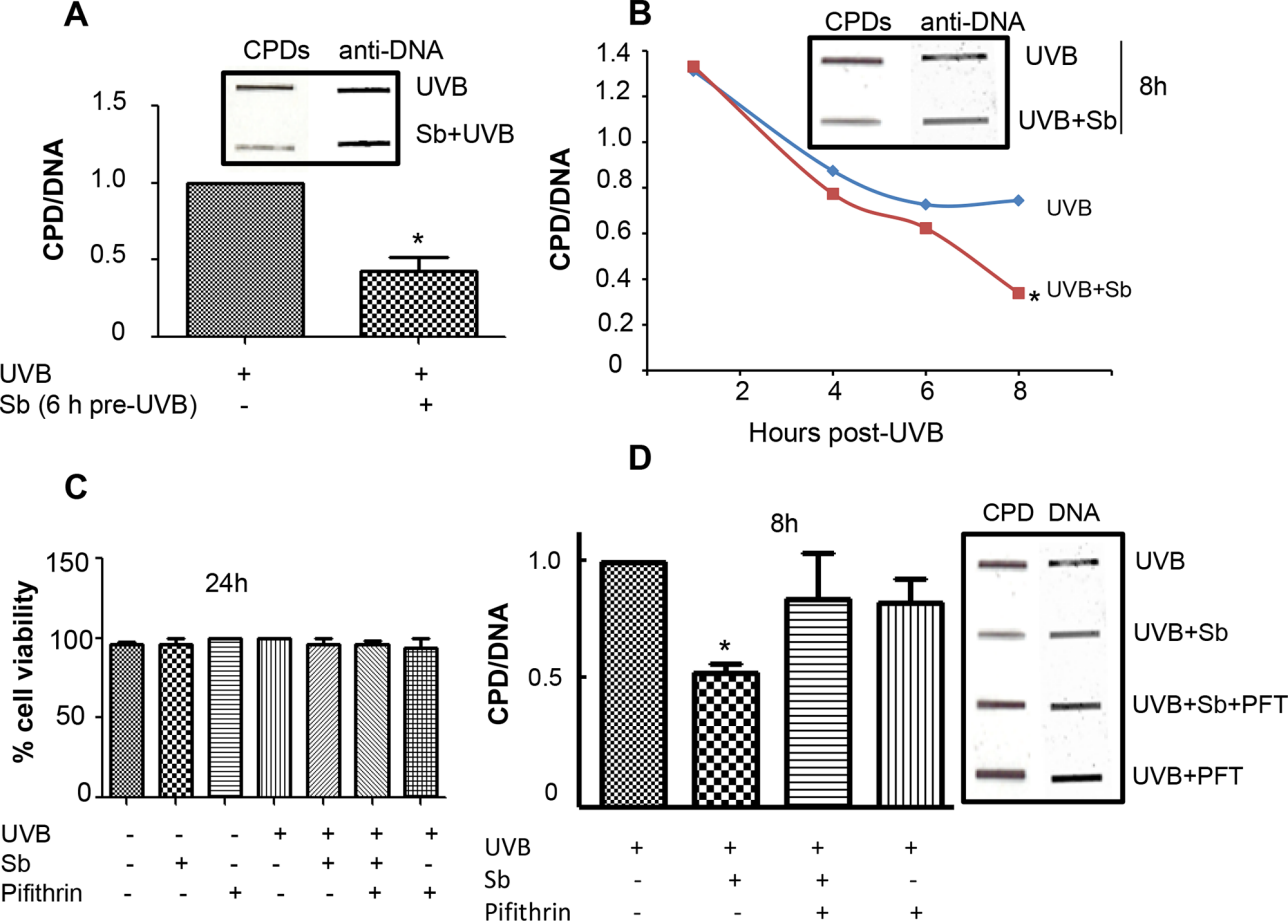
Silibinin repairs UVB-induced DNA damage in NHDFs **A.** NHDFs were treated with silibinin (100 μM) for 6 hrs and then exposed to UVB (1 mJ/cm^2^). Immediately following UVB exposure, cells were trypsinized and cell pellets collected for DNA extraction. Slot blot was performed to detect CPDs; thereafter, membranes were stripped and reprobed with single stranded DNA antibody and bands were visualized using Odyssey IR detection system. **B.** NHDFs were exposed to 1 mJ/cm^2^ UVB, treated with silibinin (100 μM) and following an appropriate incubation period (1, 4, 6 and 8 hrs), cells were collected and analyzed for CPDs by slot blot. **C.** UVB, silibinin and/or pifithrin did not affect the viability of NHDFs. NHDFs were treated with silibinin (100 μM) and/or pifithrin (7 μM) with or without UVB (1 mJ/cm^2^) exposure for 24 hrs and cell viability was assessed. The graph represents % viable cells mean ± SEM of three samples. **D.** NHDFs cells were exposed to 1 mJ/cm^2^ UVB, and immediately treated with silibinin (100 μM) ± pifithrin (7 μM) for 8 hrs and analyzed for CPDs by slot blot. Experiments were repeated at least three times and band intensity was quantified with Image J software. In each case, graph represents the amount of CPDs normalized to the control cells (UVB-treated cells) and presented as mean ± SEM of 3 samples. *; *p* < 0.05. Abbreviations: Sb: Silibinin; CPD: Cyclobutane pyrimidine dimers.

First, we optimized the UVB-dose that causes reparable DNA damage in NHDFs. Interestingly, we could not apply UVB doses (50–100 mJ/cm^2^) previously used in JB6 mouse epidermal cells [23, 24], as NHDFs underwent excessive DNA damage and were unable to repair the damage. We reduced the UVB dose gradually and found that when exposed to a 1 mJ/cm^2^ UVB dose, NHDFs did not die and were able to repair DNA damage. Henceforth, a 1 mJ/cm^2^ UVB dose was selected for all subsequent experiments. Next, we analyzed the effect of silibinin (100 μM) treatment pre- or post-UVB (1 mJ/cm^2^) exposure on DNA damage (CPDs) repair in NHDFs. As shown in Figure 1A, silibinin treatment 6 hrs prior to UVB exposure reduced CPD levels by 50% (*p* < 0.05). Next, cells were exposed to UVB and immediately treated with silibinin for 4, 6 and 8 hrs. Post-UVB silibinin treatment also resulted in an accelerated DNA repair response with a 55% (*p* < 0.05) reduction in CPD levels compared to UVB alone after 8 hrs (Figure 1B).

Previous experiments from our laboratory have consistently indicated that silibinin treatment accelerates the repair of CPDs caused by UVB radiation in both JB6 mouse epidermal cells and in SKH-I mouse skin, and that the mechanism responsible for this faster CPD removal was regulated by p53 and GADD45α increase [21, 23]. Accordingly, we next determined if p53 activity was correlated with the observed reduction in CPDs with silibinin treatment in NHDFs. For this, we used the p53 inhibitor pifithrin-α. Prior to assaying with pifithrin-α, we tested the cytotoxicity of our treatments on NHDFs cells, and found that cell viability was not significantly decreased by 1 mJ/cm^2^ UVB dose, silibinin (100 μM) and/or pifithrin-α (7 μM) even after 24 hrs of treatment (Figure 1C). Thus, we proceeded with our experiments and cells were exposed to UVB and immediately treated with DMSO, silibinin (100 μM), silibinin (100 μM) + pifithrin-α (7 μM) or pifithrin-α (7 μM) alone for 8 hrs. DNA was extracted from the cells and evaluated by slot blot method. As shown in Figure 1D, post-UVB silibinin treatment for 8 hrs significantly reduced CPD levels compared to UVB exposure alone; however, silibinin-activated DNA damage repair was completely blocked in the presence of pifithrin-α. Cells treated with pifithrin-α alone did not have significantly different CPD levels (Figure 1D). These results further supported our previous findings that p53 is indispensable for the DNA repair efficacy of silibinin. All the data in Figure 1A, 1B & 1D for CPD levels also showed equal DNA loading employing anti-ss DNA antibody.

### Silibinin increases p53 and GADD45α expression in UVB-exposed NHDFs

**Figure 2:**
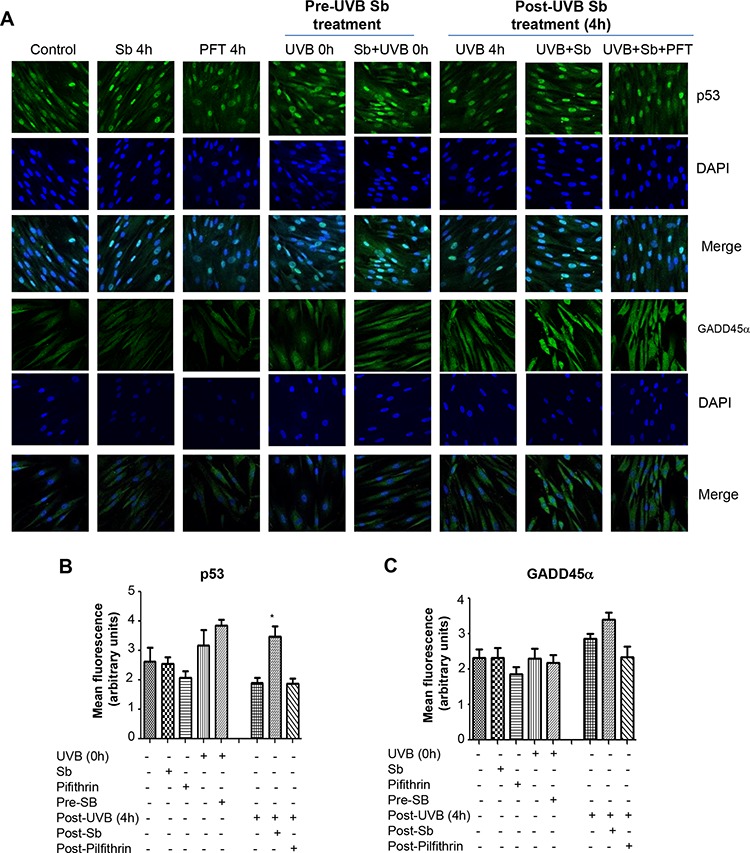
Effect of silibinin treatment on p53 and GADD45α following UVB exposure in NHDFs **A–C.** NHDFs were plated on sterile coverslips and divided into two groups: pre- and post-UVB treatment. In the pre-UVB treatment group, cells were treated with silibinin and/or pifithrin for 4 hrs, then washed and exposed to UVB 1 mJ/cm^2^. Treatment with silibinin or pifithrin alone served as relevant controls. In the post-UVB treatment group, cells were exposed first to UVB 1 mJ/cm^2^ and then immediately treated with silibinin and pifithrin for 4 hrs. At the end of the described treatments, cells were fixed and processed for confocal microscopy as detailed in the methods. Images were captured at 600x magnification on a Nikon inverted confocal microscope using 488/405 nm laser wavelengths to detect green (p53 or GADD45α) and blue (DAPI) emissions. Three independent experiments were performed in duplicates. Ten pictures were captured from each slide and representative pictures are presented. Images were also processed with Image J software and the bar graph represents the mean cell fluorescence ± SEM of ten measurements from a representative experiment. *; *p* < 0.05.

After observing that the DNA repair effects of silibinin were lost when p53 was inhibited, we next investigated the effect of silibinin on p53 and its downstream effectors (GADD45α and p21) in UVB-exposed NHDFs. As shown in Figure 2A and 2B, p53 expression was increased in UVB-exposed NHDFs compared to basal p53 expression in UVB-unexposed cells. Silibinin treatment of UVB-unexposed cells did not increase p53 expression; however, silibinin treatment prior to UVB exposure did increase p53 levels in NHDFs, though not statistically significantly (Figure 2A and 2B). Silibinin treatment for 4 hrs following UVB-exposure resulted in a significant increase in p53 expression that was completely eliminated in the presence of pifithrin-α (Figure 2A and 2B). Next, we evaluated the expression of the p53 effectors, GADD45α and p21, as these molecules are also involved in DNA repair and in addition, confirm p53 activation. As shown in Figure 2A and 2C, silibinin treatment pre- or post-UVB exposure increased GADD45α levels, which was decreased in the presence of pifithrin-α but these changes were not statistically significant. A similar pattern in p21 expression was also observed following silibinin treatment pre- or post-UVB exposure in NHDFs (data not shown).

### Silibinin activates NER machinery in UVB-exposed NHDFs

**Figure 3:**
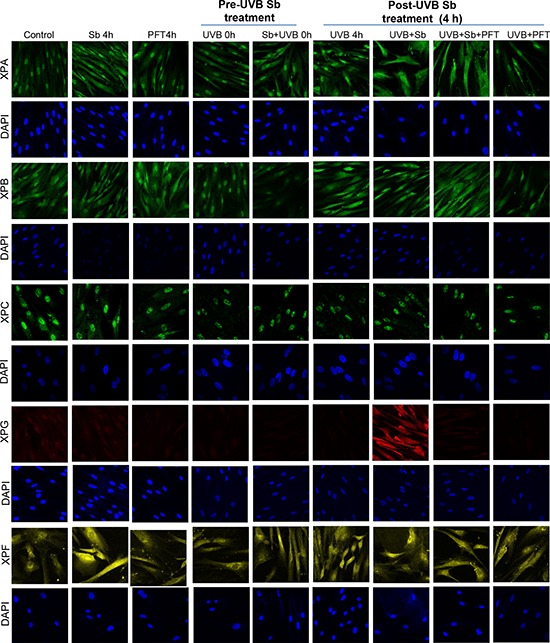
Silibinin activates the NER pathway in UVB-exposed NHDFs NHDFs were plated on sterile coverslips divided into two groups: pre- and post-UVB treatment. In the pre-UVB treatment group, cells were treated with silibinin and/or pifithrin for 4 hrs, then washed and exposed to UVB 1 mJ/cm^2^. Treatment with silibinin or pifithrin alone served as relevant control. In the post-UVB treatment group, cells were exposed first to UVB 1 mJ/cm^2^ and then immediately treated with silibinin and/or pifithrin for 4 hrs. At the end of the described treatments, cells were fixed and processed for confocal microscopy as detailed in the methods. Images were captured at 600x magnification on a Nikon inverted confocal microscope using 633/561/514/488/405 nm laser wavelengths to detect red, yellow, green and blue emissions. Three independent experiments were performed in duplicates. Ten pictures were captured from each slide and representative pictures are presented.

**Figure 4:**
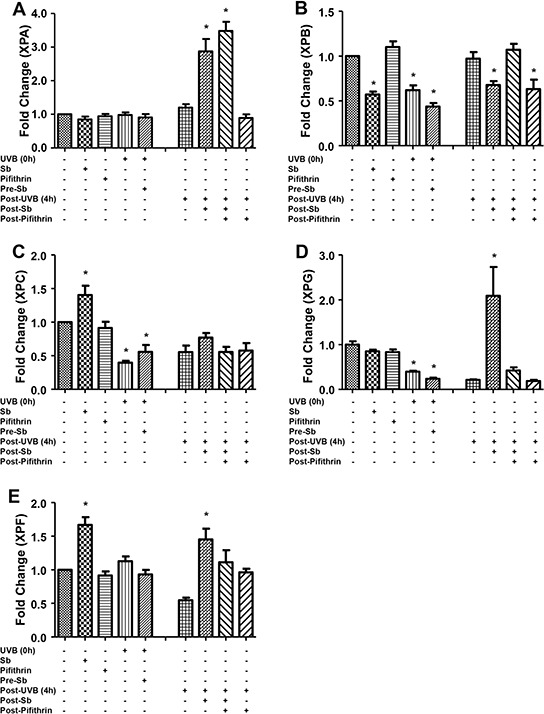
Silibinin activates the NER pathway in UVB-exposed NHDFs Images from figure 3 were processed with Image J software and the bar graph represents the mean cell fluorescence ± SEM of ten measurements from a representative experiment and normalized against the control fluorescence. *; *p* < 0.05.

Next, we examined silibinin's effect on molecules involved in the NER pathway. As shown in Figure 3 (and quantified in Figure 4), silibinin treatment post-UVB exposure strongly increased XPA levels which was not reduced in the presence of pifithrin-α; however, we observed a decrease or no significant change in the expression of XPB and XPC, respectively, under similar treatment conditions. Interestingly, we observed a strong increase in XPG and XPF expression with silibinin treatment 4 hrs following UVB exposure (Figures 3 & 4).

**Figure 5:**
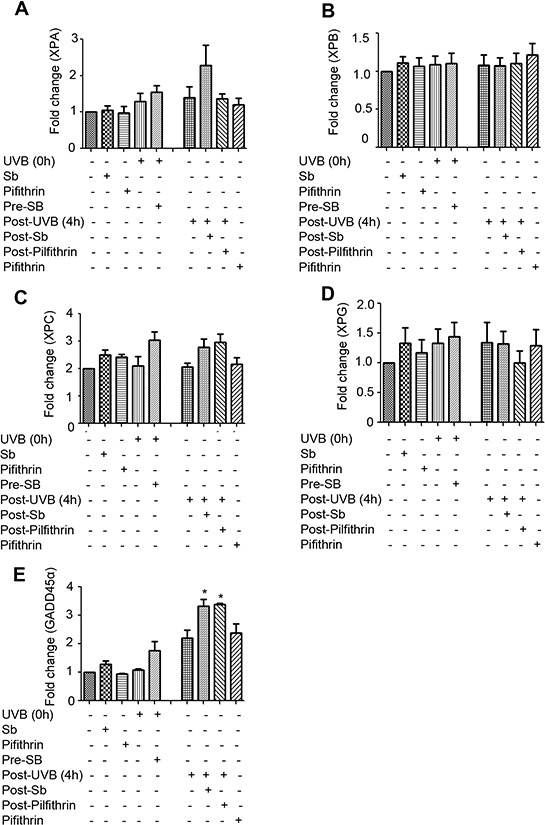
Effect of silibinin treatment on the gene expression of NER regulators and GADD45α in UVB-exposed NHDFs NHDFs were plated and divided into two groups: pre and post-UVB treatment. In the pre-UVB treatment group, cells were treated with silibinin and/or pifithrin for 3 hrs, then washed and exposed to UVB 1 mJ/cm^2^. Treatment with silibinin or pifithrin alone served as relevant controls. In the post-UVB group, cells were exposed first to UVB 1 mJ/cm^2^ and then immediately treated with silibinin and/or pifithrin for 3 hrs. At the end of the described treatments, the mRNA levels of **A.** XPA, **B.** XPB, **C.** XPC, **D.** XPG and **E.** GADD45α were measured by real-time RT-PCR as described in methods. Samples were run in triplicate, and three independent experiments were performed for every gene of interest. The mRNA level of gene was normalized to those of β-actin mRNA in each sample. *; *p* < 0.05.

Next, to understand the mechanisms underlying the observed changes in NER related molecules, we examined the effect of silibinin treatment on the mRNA levels of these molecules via RT-PCR. As shown in Figure 5A–5E, silibinin treatment 3 hrs after UVB exposure increased XPA expression which was decreased by pifithrin-α; but there was no change in the mRNA level of other NER molecules (XPB, XPC, and XPG) suggesting that post-transcriptional/translational events could be the possible mechanisms involved in their increased expression. Importantly, GADD45α mRNA expression was also significantly increased by silibinin treatment 3 hrs following UVB-exposure; however, GADD45α was not decreased in the presence of pifithrin-α suggesting the involvement of transcription factors other than p53. We also did not observe any change in the mRNA levels of DDB1, DDB2 and p53 under any of the above mentioned treatment conditions (data not shown).

### Silibinin-activated DNA repair is lost in XPA and XPB deficient cells

**Figure 6:**
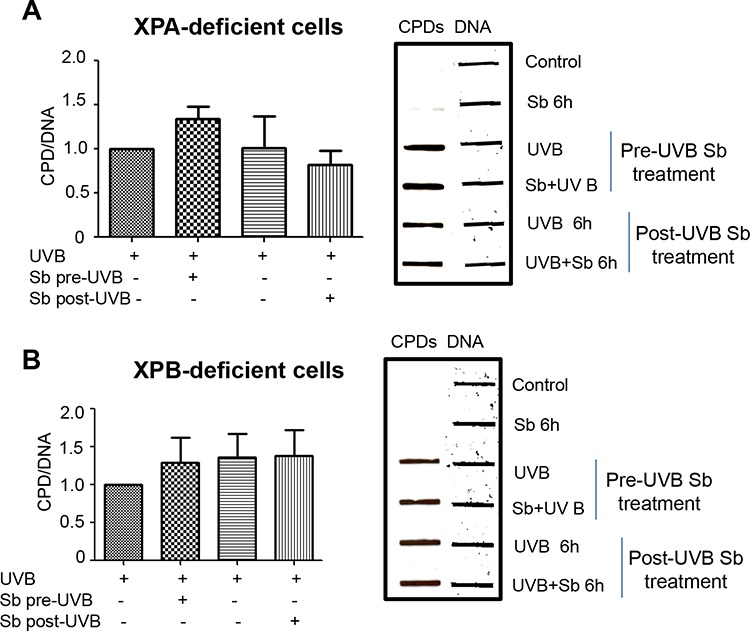
Silibinin-activated DNA repair effect is lost in XPA and XPB deficient human dermal fibroblasts **A–B.** XPA (GM05509) and XPB (GM21072) cells were either pre-treated with silibinin (100 μM) for 6 hrs followed by UVB (1 mJ/cm^2^) exposure or were exposed to UVB 1 mJ/cm^2^ and then treated with silibinin (100 μM) for 6 hrs. Cells treated with silibinin (100 μM) alone for 6 hrs served as control. Immediately after the desired treatment, cells were trypsinized and cell pellets collected for DNA extraction. Slot blot was performed to detect CPDs; thereafter, membranes were stripped and reprobed with single stranded DNA antibody and bands were visualized using Odyssey IR detection system. The graph represents CPDs normalized to the control cells collected immediately after UVB 1 mJ/cm^2^ exposure (UVB 0 hour). Bars are the mean ± SEM of three independent experiments.

Next, we assessed the effects of silibinin on dermal fibroblasts from xeroderma pigmentosum patients deficient in XPA or XPB. Silibinin treatment either 6 hrs prior to UVB-exposure or following UVB exposure showed no significant effect on CPD levels in these two XP deficient fibroblasts (Figure 6A and 6B). These results clearly suggest the important role of XPA and XPB in silibinin-activated UVB-induced DNA damage repair. These results also suggest that the DNA repair effect of silibinin treatment prior to UVB exposure is not a radiation filtering or sunscreen effect.

## DISCUSSION

The present study provides the first detailed mechanistic evidence on the activation of NER machinery and DNA repair by silibinin in UVB-exposed human dermal fibroblasts. These findings are important because silibinin not only increased the spatial-temporal presence of NER factors in UVB exposed cells but also stimulated mRNA production of some of the factors involved in DNA damage repair. These results also suggest the preventive efficacy of silibinin as its pre-treatment reduced UVB-induced DNA damage, probably through conditioning the cells for a faster NER response rather than a sunscreen effect. Furthermore, our results also suggested the important role of p53 and GADD45α in the DNA repair effects of silibinin via regulating several components of the NER pathway. This is in tandem with a recently published study where we reported that silibinin treatment promoted UVB-induced DNA damage repair via p53 and GADD45α activation in JB6 cells by inducing a cell cycle arrest [23]. Consistent with this, it has been reported by us that silibinin has strong chemopreventive effects against UVB-induced photocarcinogenesis *in vivo* in p53 wild type mice [21, 25–27]. More importantly, recently we observed that silibinin failed to prevent UVB-induced skin tumors in p53 knock-out mice (our non-published observations); further supporting the findings that p53 is necessary for the photodamage repair effects of silibinin. Currently, we are characterizing the effect of silibinin on NER pathway following UVB-exposure in both p53 wild type and p53 knock-out mice.

In NER machinery, the XPC protein is considered indispensable for DNA damage recognition [28], and it has been reported that p53 activates XPC in response to DNA damage [16]. Wang *et al*. reported that the total cellular levels of XPC and XPB were similar in both p53-wild type and p53-null cells but p53 played a pronounced role in the damage recognition and subsequent assembly of repair machinery during GGR [14]. In another study, the same research group examined p53 role in XPC induction in response to DNA damage in p53-wild type and p53-null cells, and found that the XPC DNA damage recognition factor is controlled transcriptionally by p53 through its downstream protein DDB2 [29]. They found that the UV radiation-induced redistribution of XPC was equally compromised in p53-deficient as well as DDB2-deficient human cells [29]. When they restored DDB2 function, the recruitment of XPC to DNA damage sites *in situ* was enhanced as well as the global repair of CPDs [29]. Results from the present study showed that XPC expression was slightly increased by silibinin in UVB-exposed NHDFs; however, silibinin treatment did not increase DDB2 mRNA transcript after UVB exposure. There could be a possibility that DDB2 is activated earlier than the time-point we studied (3 hrs), suggesting the need for a detailed time-course study to further establish silibinin's effect on key molecules involved in DNA damage recognition following UVB exposure.

XPB helicase, as a part of the TFIIH complex, is involved in the unwinding of the DNA complex at the site of damage. TFIIH contains a total of ten subunits, four of which have detectable enzymatic activities. Cdk7 possesses RNAPII kinase activity, both XPB and XPD have DNA helicase and ATPase activities and p44 has ubiquitin ligase activity. XPB has been reported to have roles in the NER pathway as well as in transcription [30]. It has been shown that p53 can bind to the TFIIH helicase sub-units, XPB and XPD, and modulates their helicase activity. Other reports have pointed out that the XPB and XPD helicases are also components of the p53 initiated apoptotic pathway [31, 32]. Wang *et al*. performed co-localization experiments on NER factors which showed that in normal human cells, XPC and TFIIH were rapidly recruited to damaged DNA [14]. On the other hand, the recruitment of XPC and TFIIH to damaged DNA was considerably less efficient in p53-null or p53-compromised cells [14, 32]. Our results clearly showed that the DNA repair efficacy of silibinin was completely compromised in XPB-deficient fibroblasts. However, further studies are warranted to understand silibinin's effect on other key molecules in the TFIIH complex and their role in the DNA repair efficacy of silibinin.

The importance of the XPA factor is evident in patients harboring mutations in the gene as they have a skin cancer prone phenotype; and also XPA −/− mice are prone to skin tumors development [33]. Li *et al*. examined the effect of the transcriptional function of p53 on XPA nuclear import in A549 cells which were pre-incubated with pifithrin-α before the UV treatment [34]. They reported that pifithrin-α significantly reduced UV-induced XPA nuclear import as compared to DMSO-treated cells [34]. Their data suggested that p53 may regulate control UV-induced nuclear import of XPA through its transcriptional activity [34]. Results from our present study also showed that silibinin increases XPA expression in UVB-exposed NHDFs. Moreover, the DNA repair efficacy of silibinin was compromised in XPA-deficient fibroblasts. These results clearly suggest that XPA is critical to the DNA damage repair induced by silibinin.

XPG is an endonuclease and it is indispensable for the operation of NER machinery. XPG cleaves the DNA strand at the 3′ extreme of DNA damage, and XPG binding stabilizes the pre-incision complex and it is essential for 5′ end cleavage by the ERCC1/XPF endonuclease [11, 35–37]. It has been shown that the recruitment of XPG requires functional TFIIH and that the binding of XPG to damaged DNA does not require DDB2 [38]. XPG has been reported to suppress UV-induced apoptosis in human fibroblasts [38]. Also, it plays role in the transcription by RNA polymerases I and II [39, 40] and in the recruitment of the PC4 (Human positive cofactor 4) transcription factor to DNA lesions [41]. XPG needs XPA and RPA to be present to perform the 3′ incision and the physical presence of XPG is required for the 5′ incision activity of ERCC1-XPF on damaged DNA [35, 36]. Our results showed that silibinin treatment strongly increased the XPG and XPF protein expression when applied after UVB exposure. However, the mRNA level of XPG and XPF was not significantly changed by silibinin suggesting the need to further understand silibinin's effect on post-translational mechanism/s involved in the stabilization of these proteins following UVB-exposure in NHDFs.

Taken together, these novel findings clearly suggest that silibinin exerts its DNA damage repair effects through activating multiple players involved in the NER pathway, and that this effect is mostly as a result of p53 activity, as silibinin's efficacy was severely compromised when p53 was inhibited. Overall, these results, together with our others recently published studies, further support silibinin's usage as a potential inexpensive, effective, and non-toxic agent for skin cancer chemoprevention.

## MATERIALS AND METHODS

### Reagents

Silibinin (purity >98%) and puromycin were purchased from Sigma-Aldrich (St. Louis, MO), pifithrin-α was obtained from Calbiochem (Temecula, CA). p53, p21, GADD45α, XPA, XPB, XPC, and XPG antibodies and normal goat serum were from Santa Cruz Biotechnology (Santa Cruz, CA). Horseradish peroxidase (HRP)-labeled anti-thymine dimer antibody was from Kamiya Biomedical Company (Seattle, WA), and antibody to ssDNA (MAB3299) was obtained from Chemicon (Temecula, CA). Genomic DNA and mRNA purification kit, specific primers for human NER factors (XPA, XPB, XPC, XPG, DDB1 and DDB2), p53 and GADD45α as well as PCR-master mix were obtained from Qiagen (Germantown, MD). β-actin primers were obtained from Sigma (St. Louis, MO). IR800 fluorescent dye-labeled anti-mouse IgGs was from LI-COR Biosciences (Lincoln, NE). AlexaFluor 488, 594, 555 and 647 secondary antibodies were purchased from Molecular Probes Inc. (Eugene, OR).

### Cell culture

NER proficient normal human dermal fibroblasts (GM08399) (NHDFs), human NER-deficient XPA dermal fibroblasts (GM05509), and XPB dermal fibroblasts (GM21072) were obtained from Coriell Institute (National Institute of General Medical Sciences, Human Genetic Cell Repository). All fibroblasts were authenticated by the Coriell Institute (Camden, NJ). Cells were grown in minimum essential medium supplemented with 2 mM L-glutamine and 10% fetal bovine serum (FBS; Gibco) in a 5% CO2 humidified incubator at 37°C. All media and additives were from Gibco (Grand Island, NY). All experiments were carried out with fibroblasts at passage number <20.

### UVB irradiation

Low passage NHDFs or the NER deficient XPA and XPB fibroblasts were grown to 80% confluency and treated with 100 μM silibinin for 4 or 6 hrs followed by UVB (1 mJ/cm^2^) exposure as detailed earlier (18). Briefly, media or silibinin treatment was aspirated and the cells were washed twice with PBS, after this a thin layer of PBS was added and cells were immediately exposed to UVB 1 mJ/cm^2^. After this, cells were treated with DMSO, silibinin (100 μM), silibinin (100 μM) + pifithrin-α (7 μM) or pifithrin-α (7 μM) for 4, 6, 8 or 24 hrs. A subset of cells was collected immediately after the UVB exposure and another group of cells were given identical treatments but sham UVB irradiated. The UVB radiation source was a bank of four FS24T12-UVB-HO sunlamps equipped with a UVB Spectra 305 Dosimeter (Daavlin Co., Bryan, OH), which emitted about 80% radiation in the range of 280–340 nm with a peak emission at 314 nm.

### Trypan blue exclusion assay

NHDFs were plated at a density of 7,000/cm^2^ in 12 well plates under normal culture conditions. Next day, NHDFs were exposed to UVB (1 mJ/cm^2^) and immediately after that were treated with DMSO, silibinin (100 μM), silibinin (100 μM) + pifithrin-α (7 μM) or pifithrin-α (7 μM). Control plates received the same treatments but sham UVB irradiated. Cells were incubated for 24 hrs, then were harvested by trypsinization, stained with Trypan blue (Gibco, Grand Island, NY) and counted for live and dead cells using a hemocytometer.

### Southwestern slot blot

After appropriate treatments, genomic DNA was isolated from cells by using DNeasy purification kit from Qiagen. The DNA was denatured by incubating with 4M NaOH and 100 mM EDTA at 90°C for 10 minutes, followed by rapid chilling on ice and adding an equal volume of 2M ammonium acetate and transferred to positively charged nitrocellulose membrane by vacuum slot blotting (Bio-Dot Apparatus; Bio-Rad, Hercules, CA). The membranes were baked for 30 min at 85°C, blocked with 5% non-fat milk and incubated overnight with HRP labeled-anti-CPD antibody, and bands detected by chemiluminescence. Membrane was stripped and re-probed against single-stranded DNA; IR800 fluorescent dye-labeled second antibody was used and DNA loading control bands were visualized in LI-COR Odyssey.

### Immunofluorescence

Expression of the NER factors and other DNA damage repair regulators (p53, p21 and GADD45α) was analyzed by immunofluorescence using a confocal microscope. Briefly, NHDFs were seeded onto coverslips, and next day, cells were treated as mentioned above. At the end of the desired treatments, cells were washed and fixed in 4% formaldehyde for 30 minutes at room temperature. After washing with PBS, cells were treated with methanol for 10 minutes at −20°C, then washed again with PBS and blocked with normal goat serum and FBS for one hr at room temperature. After this, cells were incubated overnight at 4°C with primary antibodies for p53, p21, GADD45α, XPA, XPB, XPC and XPG. Cells were then washed with PBS and incubated with specific secondary antibody and DAPI for 60 min. Thereafter, cells were washed in dark and coverslips were mounted for analysis. Cell images were captured at 600x magnification on a Nikon inverted confocal microscope using 633/561/514/488/405 nm laser wavelengths to detect red, yellow, green and blue emissions. Images were processed with ImageJ software.

### Real-time reverse transcription-PCR

NHDFs were exposed to UVB with or without the pre/post treatments with DMSO, silibiin, silibinin + pifithrin-α or pifithrin-α. At the end of the desired treatment, cells were harvested and total RNA was isolated using RNeasy kit from Qiagen (Germantown, MD). Quantification of RNAs was performed in a Nanodrop 2000 from Thermo scientific (Wilmington, DE). Genomic DNA was eliminated, and first-strand cDNA were prepared using RT2^−^PCR first strand kit (Qiagen). The mRNA levels for the human NER factors (XPA, XPB, XPC, XPG, DDB1 and DDB2), p53 and GADD45α were measured by real-time quantitative reverse transcription-PCR using ABI 7900HT Fast System from Life technologies (Grand Island, NY) at the Molecular Biology Core Facility of the University of Colorado Cancer Center. The final reaction volume was 25 μl including 2 μl of cDNA. The standard PCR amplification conditions were: 95°C for 10 min, then 40 cycles at 95°C for 15 sec and 60°C for 1 min, as per vendor's protocol. Samples were run in triplicate, and three independent experiments were performed for every gene of interest. The mRNA level of genes was normalized to those of β-actin mRNA in each sample. Calculations for determining the relative level of gene expression were made using the ΔΔCT method of quantification as reported by Livak *et al*. [42].

### Statistical analysis

Statistical analysis was performed using GraphPad Prism version 5 (San Diego, CA) for Windows. Data were analyzed using analysis of variance (ANOVA) followed by Tukey's test as post-test and a statistically significant difference was considered at *P* < 0.05.

## Abbreviations

BCC: Basal cell carcinoma
CS: Cockayne's syndrome
CPDs: Cyclobutane pyrimidine dimers
DDB: DNA damage-binding protein
DMSO: Dimethyl sulfoxide
GGR: Global genomic repair
NER: Nucleotide excision repair
NHDFs: Normal human dermal fibroblasts
NMSCs: Non-melanoma skin cancers
PCNA: Proliferating cell nuclear antigen
6-4 PPs: 6-4 photoproducts
Sb: Silibinin
SCC: Squamous cell carcinoma
TCR: Transcription-coupled repair
TFIIH: Transcription factor IIH
UVB: Ultraviolet radiation B
XP: Xeroderma pigmentosum
